# Biobased Boston Ivy‐Inspired Cryogel Integrating Radiative Cooling and Phase Change Energy Storage for Adaptive Thermal Regulation

**DOI:** 10.1002/advs.78323

**Published:** 2026-10-15

**Authors:** Sining Huang, Zhiguo Li, Yang Liu, Chengyu Wang, Yi Lu, Erlantz Lizundia, Siqi Huan

**Affiliations:** ^1^ State Key Laboratory of Utilization of Woody Oil Resource Northeast Forestry University Harbin People's Republic of China; ^2^ Horticultural Branch Heilongjiang Academy of Agricultural Sciences Harbin Heilongjiang People's Republic of China; ^3^ Key Laboratory of Bio‐based Material Science & Technology (Ministry of Education) Northeast Forestry University Harbin People's Republic of China; ^4^ Institute of Process Engineering Chinese Academy of Sciences Beijing People's Republic of China; ^5^ Life Cycle Thinking Group, Department of Graphic Design and Engineering Projects, Faculty of Engineering in Bilbao University of the Basque Country (EHU) Bilbao Spain

**Keywords:** biobased material, cryogel, nanocellulose, phase change energy storage, Pickering emulsion, radiative cooling

## Abstract

Boston ivy is a traditional plant for building heat management through shading and transpiration. Inspired by its “vine‐leaf” system, we design a bionic cryogel that functionalizes daytime cooling and nighttime thermal buffering, promoting sustainable and comfortable housing and living environments. The aligned channels mimic vine tendrils, delivering an ultralow radial thermal conductivity (0.035 W m^−1^·K^−1^), high solar reflectance (95.28%), and high infrared emissivity (97.04%). Embedded phase‐change materials (PCMs) present leaf‐like, functionalized structures that store excess solar energy during the daytime and release latent heat at night, achieving 6.2°C sub‐ambient cooling under sunlight. In darkness, the stored latent heat is released, keeping the cryogel 2.4°C above ambient and preventing nocturnal overcooling. This spatiotemporal synergy between daytime radiative cooling and nighttime heat release enables climate‐adaptive thermal management, reducing the urban heat buildup with no need for electricity consumption. Our bionic design shifts thermal regulation from passive adaptation to active thermal planning using low carbon footprint materials as proven by life cycle assessment (LCA), offering a more sustainable pathway for energy‐efficient buildings.

## Graphical abstract

A bionic cryogel containing phase‐change materials that provides daytime radiative cooling and nighttime heat release is developed to promote environmentally sustainable and comfortable housing and living environments with reduced building energy demand. 6.2°C sub‐ambient cooling under sunlight is achieved, while in darkness, the stored latent heat is released, keeping the cryogel 2.4°C above ambient and preventing nocturnal overcooling.

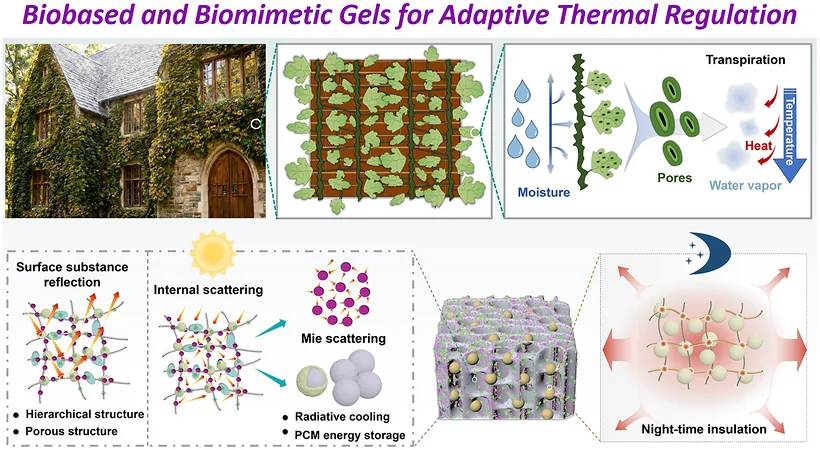

## 1 Introduction

The building sector accounts for more than 30% of global energy consumption, and this energy is largely used for heating, ventilation, air conditioning, and refrigeration [1, 2]. As extreme temperature events have become more frequent worldwide in recent years [3], developing low‐cost, low‐energy cooling technologies has emerged as an urgent need for building a sustainable society. Passive radiative cooling (PRC) was proposed in the 1970s as an electric‐free approach that uses solar energy to maintain indoor thermal comfort by dissipating heat through the atmospheric window (8–13 µm) to cold outer space [4, 5].

Effective PRC demands two critical optical properties, that are (i) high solar reflectance to minimize heat gain and (ii) high infrared emissivity to maximize heat loss through the atmospheric window. Various forms of PRC materials, including films, coatings, paints, cryogels, glass, and textiles, have demonstrated sub‐ambient cooling capabilities under clear daytime skies for diverse applications [6, 7, 8, 9, 10, 11, 12, 13, 14, 15]. However, PRC alone cannot provide reliable, all‐weather thermal regulation without additional energy input. Its strong cooling effect is optimal under clear skies, but two major drawbacks persist: (i) nighttime overcooling, (ii) suboptimum performance under cloudy or humid conditions. These limitations motivate the development of hybrid strategies that combine PRC with complementary thermal management mechanisms for all‐weather thermal regulation.

In direct contrast to PRC materials, phase change materials (PCMs) offer a complementary heat storage and release function by absorbing and releasing latent heat through solid‐liquid phase transition. This provides a thermal buffer against sudden temperature fluctuation, maintaining stable temperature control over day‐night cycles [16]. However, the practical challenge of PCMs arises from the natural fluidity of melted PCMs after absorbing heat, compromising long‐term material stability, especially under prolonged exposure to heat waves that exceed their latent heat capacity.

PRC‐PCM hybridization offers a promising synergistic design to address the limitations of each component [17, 18, 19, 20]. During the daytime, radiative cooling reflects sunlight and emits thermal radiation into space, while the PCM absorbs solar energy that penetrates the reflective layer. This dual action not only enhances cooling performance but also suppresses degradation of PRC materials and PCM leakage issues. At night, PCMs release the stored latent heat, compensating for continued radiative loss and preventing temperatures from dropping below ambient comfort levels. Thereby, this PRC‐PCM combination complements each other temporally, creating a dynamic thermal regulation cycle for adaptive heat management.

In this work, we present a rational material architecture design for PRC‐PCMs hybridized thermal management. Inspiration originates from *Parthenocissus tricuspidata*, or Boston ivy, a traditional gardening plant native to eastern Asia used for building cooling through shading and transpiration [21, 22]. Herein, we design a bionic cryogel that integrates radiative cooling with phase‐change energy storage. Biomass fiber is selected as a platform material because, when processed into a porous structure, it offers a high refractive‐index mismatch between the material and air voids, enabling strong scattering and high thermal emissivity, thereby leading to effective radiative cooling. As cellulose is composed of biogenic carbon, its utilization in engineered applications avoids the incorporation of fossil carbon into the technosphere, which in turn limits the addition of fossil carbon into the atmosphere [23, 24]. Moreover, cellulose can be engineered through multiple top‐down processing routes [25, 26], enabling tailored structures that fit targeted applications. This material features directional pore channels (mimicking vine tendrils) and embedded phase‐change emulsion droplets (stabilized synergistically by TOCNF and PVA, acting like leaves), fabricated via freeze‐casting of a gelatin/hydroxyethyl cellulose/silica (GHS) scaffold. Such an architecture synergistically combines solar reflection, thermal storage, and thermal insulation, enabling dynamic and adaptive temperature regulation across day‐night cycles. Hydrophobic modification further imparted self‐cleaning and water resistance to enhance material durability for building applications. The resulting cryogel exhibits 95.28% solar reflectance and 97.04% infrared emissivity, achieving 6.2°C sub‐ambient cooling under sunlight and maintaining 2.4°C above ambient at night, demonstrating a more sustainable pathway for energy‐efficient buildings as proven by screening life cycle assessment (LCA) results on material utilization.

## 2 Experimental Section

### 2.1 Materials

TEMPO‐oxidized nanocellulose (TOCNF, carboxylic group content of 1.4 mmol·g^−1^, 1.28 wt.%) with a length of 1–5 µm and an aspect ratio of 200 was derived from softwood bleached sulfate pulp, supplied by Tianjin Wood Wizard Biotechnology Co., Ltd., China. Detailed morphological characterization is available in our published work [27]. Paraffin wax (PW, melting point about 26°C) was sourced from Dongguan Zhangmutou Shengbang Plastic Products Trading Department, China. Polyvinyl Alcohol (PVA, M_w_: 205000), 3‐glycidoxypropyltrimethoxysilane (KH‐560, Purity ≥ 97%), MTMS (Purity ≥ 98%) and gelatin (GA, G108396, gel strength: 240 g Bloom) were purchased from Aladdin Biotechnology Co., Ltd., China, respectively. Hydroxyethyl Cellulose (HEC, H810930) was obtained from McLean Biochemical Technology Co., Ltd., China. Hydrophilic fumed nanosilica particles (SiO_2_, Aerosil A200 F) were purchased from Evonik Degussa, Germany.

### 2.2 Preparation of Phase‐Change Material by Pickering Emulsion

Micro/nano‐scale PCM with a core‐shell structure was synthesized using Pickering emulsions as templates [28]. Paraffin wax was encapsulated in a shell formed by TOCNF and PVA, which can form a kind of complex (T/P complex). The T/P concentration in the continuous phase was optimized and fixed at 2 wt.%. Briefly, PVA was first stirred and dissolved in deionized water at 95°C for 1 h; TOCNF was then added to the PVA aqueous solution after it had cooled to room temperature, and the mixture was stirred for 30 min at 450 rpm to form the TP complex. The complex was subsequently heated to 45°C, combined with molten PW, and homogenized for 2 min (IKA‐T25, Germany) to produce the PW/T/P phase‐changed Pickering emulsion (PTP). The water‐to‐oil ratio (WOR) in the phase‐changed Pickering emulsion was adjusted to 6/4, with the optimized T/P ratio fixed at 4/6 (w/w).

### 2.3 Fabrication of Anisotropic PRC‐Assisted Phase Change Energy Storage Cryogels

The HEC/GA composite frame (GH) was prepared. The specific procedure was as follows: 2 grams of GA was dissolved in 100 mL deionized water under continuous stirring in a 65°C water bath until it became a transparent and slightly yellow solution; 1 gram of HEC was then added to the gelatin solution above, which had been cooled to room temperature, and the mixture was thoroughly blended for 30 min to complete the preparation in this part. 1 gram of SiO_2_ (silicon dioxide) and PTP (0, 0.5, 1, and 2 gram), either separately or together, was added to 25 g of the GH mixture. The resulting substances are designated as GHS and GHSPx (where x denotes the PTP dosage), respectively. Notably, the addition of SiO_2_ required the concomitant use of 1 µL of KH‐560 for cross‐linking.

Bottomless polytetrafluoroethylene (PTFE) molds were placed on a copper plate. An appropriate amount of GH, GHS, or GHSP_x_ was poured into the mold, respectively. Liquid nitrogen was poured into a copper container, and the aforementioned copper plate was placed on top of the container. This setup prevented liquid nitrogen leakage while using the copper plate's thermal conductivity to freeze the samples on its surface.

The directionally frozen samples were quickly transferred to a pre‐frozen freeze‐dryer and lyophilized at −70°C for 48 h. After removal, the samples were stored in a desiccator.

### 2.4 Hydrophobic Modification

Two beakers containing 2 mL of deionized water and 4 mL of Methyltrimethoxysilane (MTMS), respectively, were placed at the bottom of an empty desiccator. A mesh sheet was positioned above the beakers, and the GHSP_2_ sample was placed on top of the mesh. The sealed desiccator was then transferred to an 80°C oven for 10 h. Subsequently, the treated GHSP_2_ was removed and placed in a 100°C oven for 1 h to complete the hydrophobic modification. The final sample was designated as M@GHSP_2_.

### 2.5 Characterization of Anisotropic Cryogels

#### 2.5.1 Scanning Electron Microscope

Surface morphologies were observed with a scanning electron microscope (SEM, Apreo S HiVac, Thermo Scientific, USA) at 5 kV. To improve surface conductivity for observation, the samples were coated with gold using a sputtering technique.

#### 2.5.2 Wettability

Water droplets (5 µL) were applied to five separate areas of each BTC foam sample to measure the contact angle by a contact angle meter (CA‐A, Hitachi, Japan).

#### 2.5.3 Thermal Properties

Thermogravimetric analysis (TG) (STA 2500, Netzsch, Germany) was performed to evaluate thermal stability, with samples heated from 25°C to 700°C at a constant rate of 10°C min^−1^.

The thermal conductivity of different cryogels was tested under ambient conditions with a thermal conductivity analyzer (TPS 2500 S, Hot Disk, China). The anisotropic cryogel was placed on the hot plate, and the surface, internal, and hot plate temperatures over 30 min were recorded using an infrared thermal imager (FLIR E6, PerkinElmer, USA).

#### 2.5.4 PRC Performance

The reflectivity of the cryogel was measured with an ultraviolet‐visible near‐infrared (UV‐vis‐NIR) Spectrophotometer (Lambda 950, PerkinElmer, USA). The Infrared (IR) spectrometer (INVENIO‐S, Bruker, USA), equipped with an A562 integrating sphere, was utilized to measure the mid‐infrared spectral reflectivity/emissivity of the films in the wavelength range of 2.5–25 µm. Calculation formulas are provided in Equations (S1–S11). The real‐time temperature tester (AT4208, Anbai, China) monitors temperature fluctuations throughout the testing day. The ultrasonic micro‐weather station (SN‐3003‐CFSFX‐*, Saien, China) collects real‐time data on solar radiation and ambient humidity.

## 3 Results and Discussion

### 3.1 Bioinspired Design and Working Mechanism

Inspired by the vein structure of ivy leaves and their summer cooling effect [22], we presented a cross‐dimensional biomimetic design for energy‐efficient building panels. Boston ivy leaves rely on a directional vein network for mechanical support and achieve cooling through shading and transpiration. Drawing on this concept, we constructed a directional porous network within the cryogel to anchor phase‐changed Pickering emulsion particles. The aligned channels structurally resemble the ordered arrangement of leaf veins, while the anchored phase‐change particles regulate temperature through dynamic thermal storage. Although their operating mechanisms are not identical to transpiration, both achieve cooling by modulating ambient temperature through their intrinsic properties, thereby jointly fulfilling the thermal management goals, as illustrated in Figure 1a and Figure S1.

**FIGURE 1:**
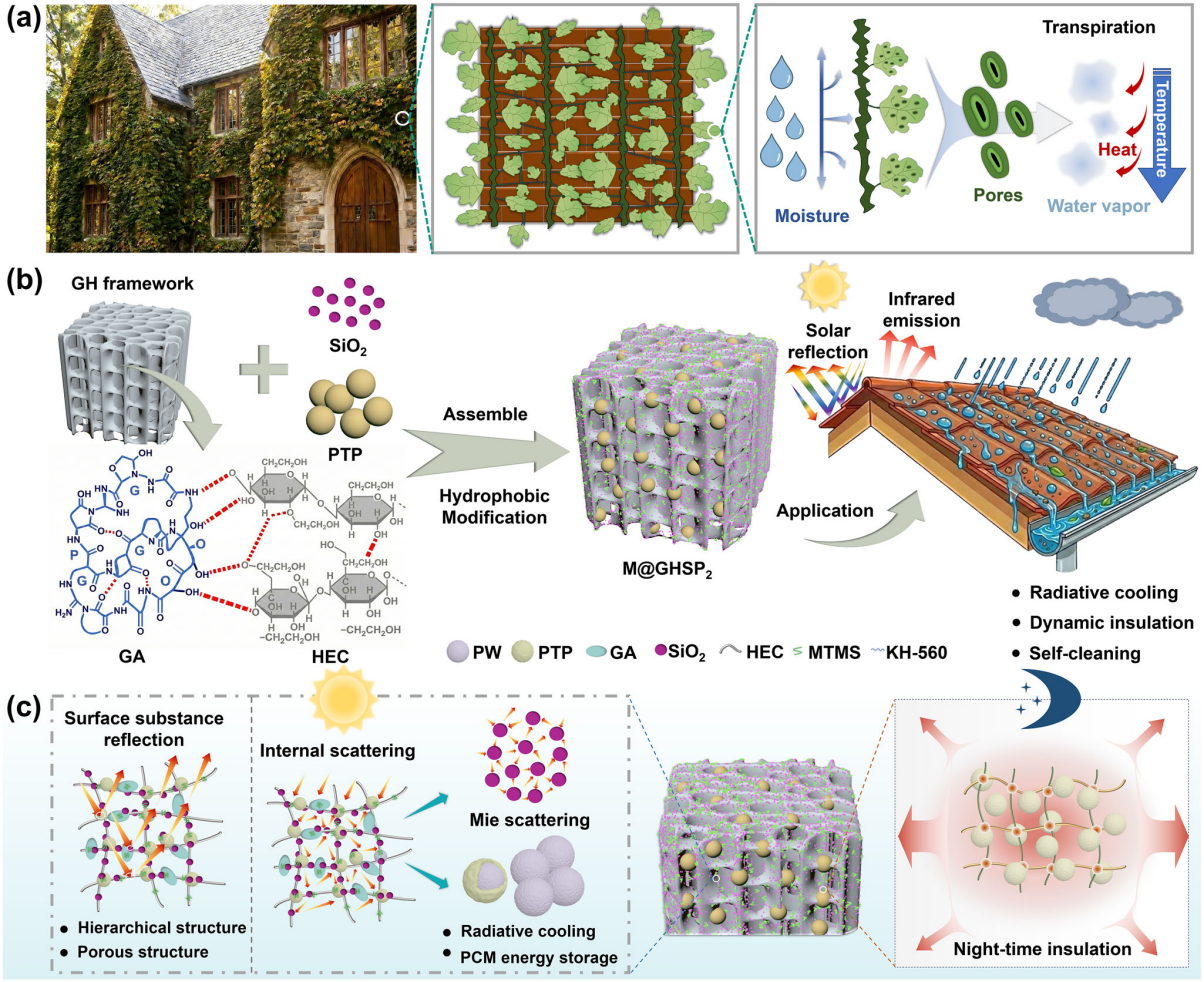
Schematic diagram illustrating the bio‐inspired design concept, material preparation process, and performance analysis. (a) Boston ivy‐inspired biomimetic concept for PRC‐PCM hybridization design. (b) Preparation process and application demonstration of the cryogel. (c) Analysis of the mechanism for PRC and phase change energy storage.

To realize this design, we first construct a three‐dimensional skeleton of gelatin and hydroxyethyl cellulose that form stable hydrogen bonds, providing a robust scaffold that mimics vine tendrils. Phase‐change emulsion particles, acting like leaves, are uniformly loaded onto this skeleton. Silica nanoparticles and PTP are later incorporated into the network, followed by hydrophobic modification with MTMS. The resulting cryogel (denoted as M@GHSP2) integrates PRC, phase‐change energy storage, and self‐cleaning capabilities. The unique internal architecture of this material enables high‐efficiency reflection and scattering of sunlight, dynamic thermal regulation in response to ambient temperature changes, and effective resistance to variable weather conditions (Figure 1b). These attributes render it highly promising and valuable for applications in building facades or roofing materials.

PTP fabrication is illustrated in Figure S2, which involves encapsulating phase change materials using a Pickering emulsion template, followed by integrating the resulting PCM‐loaded emulsion particles into a supporting scaffold. Specifically, the aqueous‐phase components, TOCNF and PVA (T/P complex), form a stable dual‐network structure via hydrogen bonding, which stabilizes the PW particles and facilitates the formation of Pickering emulsions [29]. This structure partially forms a thin film, thereby achieving hermetic sealing of the PW (Figure S3a). Based on the morphological observations of the freeze‐dried PTP by SEM, spherical emulsion particles were observed with a visible film‐like layer encapsulating their surface, indicating effective encapsulation of the PW due to the structural characteristics of the oil‐in‐water emulsion (Figure S3b). Optical microscopy further revealed that PTP particles exhibit a circular morphology, uniform dispersion without agglomeration, and an average particle size of 12.58 µm (Figure S3c,d). Furthermore, as shown in the inset of Figure S3d, the inverted emulsion exhibited no flow, indicating its gel‐like characteristics [30]. Analysis of the oscillation frequency sweep curves (Figure S3e) revealed that the storage modulus (G′) of the PTP consistently exceeded its loss modulus (G″), indicating the formation of a robust network structure and a high degree of gelation within the system [31].

The resulting M@GHSP_2_ possesses dual functionalities of PRC and phase‐change energy storage, as shown in Figure 1c. First, during daytime under sufficient sunlight, three simultaneous processes take place. The directional pore structure, combined with the attached SiO_2_ particles and the paraffin encapsulated within the transparent T/P composite, forms an efficient scattering network that converts solar thermal energy into thermal radiation (which is emitted into outer space). Concurrently, when the light enters the cryogel from the exterior, its propagation direction changes. Internal light propagation involves sequential scattering at the interfaces between SiO_2_ and paraffin‐based phase‐change emulsion particles, thereby extending the optical path length. Due to their inherent properties, SiO_2_ particles readily exhibit Mie scattering. In practice, the synergistic interaction between SiO_2_ and the phase‐change material effectively scatters sunlight, thereby enhancing the overall reflectivity of the cryogel [32].

As night falls and solar radiation diminishes, the phase‐change emulsion particles within the cryogel begin to act as energy‐storage units. Each particle absorbs and retains the heat generated during the day under high‐intensity solar irradiation, thereby providing the energy required to sustain the cooler environment throughout the night.

### 3.2 Microstructural Analysis

The design of PRC cryogels aims to achieve highly efficient PRC by integrating micro/nano‐scale porous structures with molecular‐level strategies [33]. The microstructural evolution in the cryogels was investigated by examining SEM images of GH, GHS, GHSP_2_, and M@GHSP_2_ (Figure 2). The freeze‐casting process, in combination with the shape adaptability of the biobased platform materials utilized [26], enabled the formation of hierarchically aligned pore channels throughout the cryogel structure (Figure S4). We initially engineered a three‐dimensional hierarchical structure composed of HEC and GA, with the schematic representation of the GH framework illustrated in Figure 2a.

**FIGURE 2:**
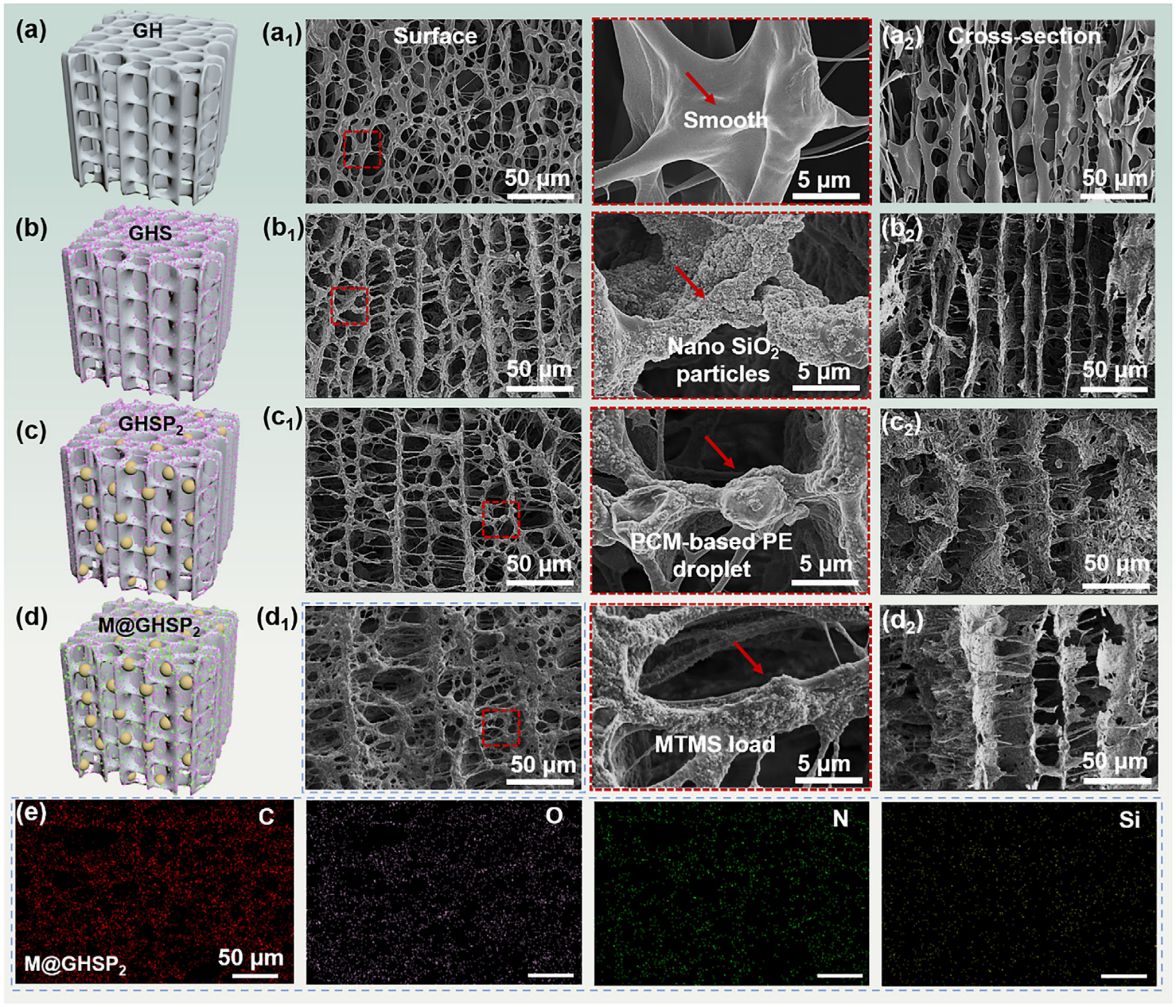
Structural characterization and elemental analysis mapping of anisotropic cryogels. Three‐dimensional schematic diagrams of the structures of (a) GH, (b) GHS, (c) GHSP_2_, and (d) M@GHSP_2_, along with scanning electron microscopy images revealing their (a_1_–b_1_) surface and (a_2_–b_2_) cross‐section microstructure.

The surface morphology of the sample exhibits uniformly distributed porous structures with smooth interfaces at the junction points, as shown in Figure 2a_1_. The cross‐sectional view revealed distinct aligned channels characteristic of directional freezing (Figure 2a_2_). Following the incorporation of nano‐SiO_2_, the cryogel's internal architecture remains intact, while detailed images confirm homogeneous nanoparticle deposition on the framework, resulting in roughened interconnected networks (Figure 2b,b_1_,b_2_). This structural evolution established a robust foundation for enhancing both solar reflectivity and infrared emissivity.

PTP was integrated into the GHS framework, exhibiting uniform dispersion and adhesion within the three‐dimensional network (Figure 2c). SEM images of GHSP_0.5_, GHSP_1_, and GHSP_2_ revealed a significant increase in the number of uniformly distributed emulsion particles within the network skeleton as PTP content increased. Notably, PW exhibits excellent PRC capability, and the selected transparent encapsulation layer (T/P complex) does not interfere with its optical interactions. The effective loading of emulsion particles was confirmed from both surface and cross‐sectional perspectives. Further examination revealed that the spherical particle surfaces were also coated with rough nanosilicates. These findings were supported by Figure 2c_1_,c_2_ and Figure S5. After MTMS modification, the cryogel's internal network structure became noticeably denser (Figure 2d).

Fourier transform infrared spectroscopy (FTIR) of the four samples confirmed the stepwise modification process (Figure S6). The original GH skeleton displays characteristic peaks of GA and HEC at 1633 cm^−1^ (C═O stretching vibration) and 1057 cm^−1^ (C─O─C stretching vibration). With the introduction of nano‐SiO_2_, an asymmetric Si─O─Si stretching vibration emerged at 1055 cm^−1^. With the addition of PTP, characteristic peaks of PW appeared at 2915 and 2848 cm^−1^, corresponding to the stretching vibrations of ─CH, ─CH_3_, and ─CH_2_ groups, respectively [34], confirming the effective loading of PTP particles. The infrared spectrum of M@GHSP_2_ obtained by hydrophobic modification of GHSP_2_ via MTMS vapor deposition revealed peaks at 1269 and 1027 cm^−1^, corresponding to the symmetric bending vibration of Si─CH_3_ and the asymmetric stretching vibration of Si─O─C, respectively. This further demonstrated the formation of siloxane networks, thereby enhancing the hydrophobicity of the material surface [35]. Elemental mapping of M@GHSP_2_ confirmed successful MTMS modification through the detected homogeneous distribution of C, N, O, and Si (Figure 2e).

Experimental observations in Figure 2 demonstrate the effective integration of nano‐SiO_2_ and PTP within the GH framework. Freeze‐casting generated structurally aligned microchannels of 2–28 µm, while MTMS vapor deposition formed a conformal hydrophobic siloxane network, establishing a material platform for subsequent cryogel functionalization.

### 3.3 Thermal Properties, Pore Structure and Hydrophobicity

It is well stablished that the thermal insulation properties depend heavily on the internal pore‐channel structures [36]. Anisotropic cryogels prepared by directional freezing exhibit different thermal conductivities in the axial and radial directions. As shown in Figure 3a and Figure S7a, the axial thermal conductivity of the four cryogels was significantly higher than the radial thermal conductivity. Among them, M@GHSP_2_ demonstrated the highest axial thermal conductivity of 0.064 W m^−1^ K^−1^, while its radial thermal conductivity was only 0.035 W m^−1^ K^−1^. This phenomenon occurs because the channels parallel to the direction of ice crystal growth after directional freezing remain unobstructed, creating air‐filled pathways. However, when the heat flows perpendicular to the ice growth direction, it must trespass numerous successive cryogel skeleton networks. The series connection of these extensive networks ultimately formed a denser pathway for heat transfer compared to the axial direction. In contrast, the isotropic cryogels prepared by conventional freeze‐drying generally exhibit higher thermal conductivity, stabilizing at approximately 0.05 W m^−1^ K^−1^ (Figure S7b).

**FIGURE 3:**
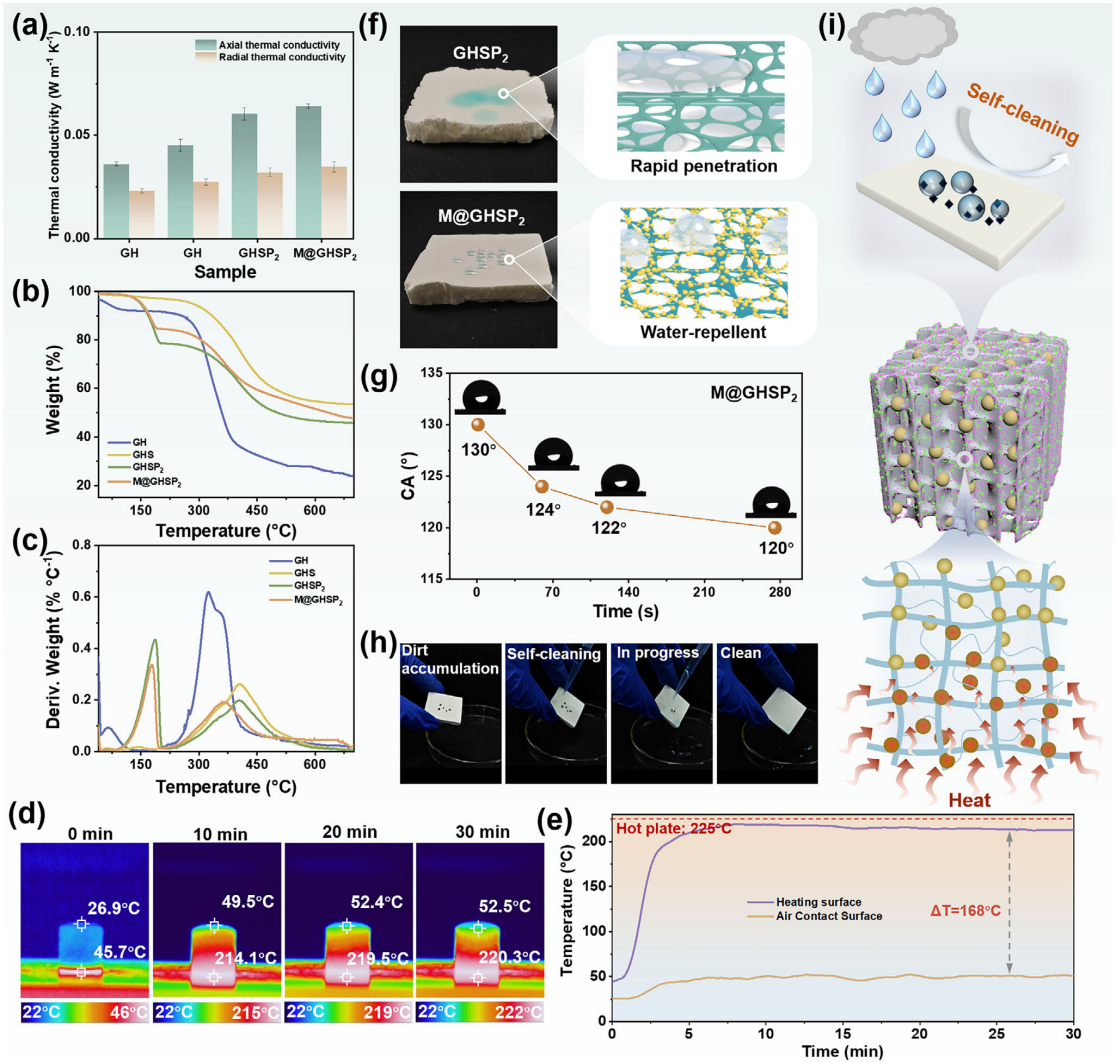
Analysis of thermal properties and surface hydrophobicity. (a) Thermal conductivity. Thermal stability analysis via (b) TGA and (c) DTG for anisotropic cryogels. (d) Temperature monitoring of the air contact and heating surface when the cryogel sample (height × diameter: 2 cm × 2 cm) was settled on the 225°C hot plate for 30 min, and (e) its corresponding temperature change curve diagram. (f) Methylene blue‐stained water droplets on the surfaces of GHSP_2_ and M@GHSP_2,_ and the schematic diagram of the relationship between microstructure and wettability of cryogel. (g) Water contact angle of M@GHSP_2_ and the captured droplet image at the corresponding time. (h) Self‐cleaning capability test of M@GHSP_2_. (i) Schematic of heat transfer conditions and self‐cleaning in microporous cryogel.

The porosity degree of the cryogels is presented in Figure S8a, as calculated by Equation (1). The porosity (*ε*) of the cryogels was calculated using the following equation:

(1) $$\varepsilon \left(\%\right)=\left(1-\frac{\rho_{b}}{\rho_{s}}\right)\times 100\%$$

where *ρ*_b_ is the bulk density of the cryogel, determined by measuring the mass and geometric volume of the sample, and *ρ*_s_ is the skeletal density, which is determined by calculating the density and the corresponding mass percentage of each component that constitutes the cryogel skeleton.

The pristine GH skeleton exhibited the highest porosity of 97.8%. The porosity gradually decreased with successive layers of the composite, reaching 92.5% when the PTP addition was maximized. The subsequent MTMS modification further reduced the porosity to 91.8%, a reduction attributable to the formation of numerous siloxane networks on the surface and within the cryogel. These networks densify the internal channels to some extent, leading to a slight decrease in porosity. Brunauer–Emmett–Teller (BET) porosity data obtained by N_2_ adsorption desorption isotherm analysis further revealed a microporous pore structure within the cryogels. The specific surface area of M@GHSP_2_ was approximately 22.26 m^2^ g^−1^ (Figure S8b), with the primary pore size distribution ranging from 2 to 10 nm, indicating the predominance of mesopores in the cryogel.

The thermal stability was investigated by thermogravimetric analysis (TGA) (Figure 3c,d and Figure S9). According to the TGA and DTGA results, all cryogels exhibited an initial mass loss between 45°C and 150°C, corresponding to the evaporation of physically adsorbed water. The TGA curve of the pure GH skeleton showed rapid decomposition up to 300°C, and then stabilized after 500°C. The corresponding DTGA curve showed a pronounced decomposition peak in the range of 280°C–350°C, attributed to the decomposition of the hydroxyethyl cellulose‐gelatin composite. The residual mass at 700°C was 23.96%. After incorporating nano‐silica into the GH skeleton, the thermal stability of the resulting composite cryogel was significantly improved. According to the DTGA curve of GHS, the residual mass at 700°C was as high as 53.77%, significantly greater than that of the GH skeleton. This enhancement is primarily attributed to the inherent rigidity and thermal stability of the nano‐silica [37]. With the incorporation of PTP, a broad peak emerged in the DTGA curve within the temperature range of 300°C–450°C, attributable to the distinctive decomposition behavior of the introduced PVA [38]. As the PTP content increased, PW volatilization reduced the sample's final residual carbon content. After hydrophobic modification, the MTMS layer acted as a physical barrier, delaying the thermal decomposition of the internal polymers. Furthermore, due to the increased inorganic content, the final residual carbon content of the modified sample was approximately 47.71%, an increase of about 1.92% over the unmodified sample (45.79%). The above analysis collectively demonstrated that the cryogels prepared in this system exhibit excellent thermal stability.

To evaluate the thermal insulation capability of M@GHSP_2_, a cylindrical sample was placed on a hot plate, and the temperature changes on the upper surface of the sample and at the hot plate contact interface were monitored in real‐time (Figure 3d,e). Based on the thermal imaging and temperature variation curves, when the hot plate was heated from room temperature to 225°C, the upper surface temperature of the sample increased from 26.9°C to 49.5°C within the first ten minutes, after which the temperature rise slowed and eventually stabilized at 52.5°C. In contrast, the bottom surface temperature rose rapidly from 45.7°C to 209.7°C within five minutes, and then increased gradually before stabilizing at 220.3°C. After 30 min, the temperature difference between the upper and lower surfaces reached up to 168°C, further confirming the material's remarkable thermal insulation capability [39, 40, 41].

The phase‐change energy storage capacity of the cryogels was verified by differential scanning calorimetry (DSC) (Figure S10). When the PTP addition was 2 g, the proportion of the phase change material (PW) in the entire system was only 22.5%, yet the latent heat of GHSP_2_ reached a maximum value of 67.69 J g^−1^. After hydrophobic modification, the PW content remained virtually unchanged at approximately 22.1%, and the latent heat of M@GHSP_2_ was largely unaffected, registering at 67.02 J g^−1^. In addition, M@GHSP_2_ exhibited excellent thermal cycling stability, with a latent heat retention of 94.37% after 100 thermal cycles (Figure S10c and Table S1). It is noteworthy that when the PTP addition exceeded 2 g, the emulsion's high viscosity resulted in inadequate mixing during the composite process, thereby compromising the formation of a highly ordered structure during directional freezing. Therefore, a PTP dosage of 2 g was selected as the optimal sample for the subsequent experiments. Leakage resistance is a critical metric for evaluating high‐performance phase change materials. To assess this property, both GHSP_2_, the sample with the highest PTP content, and the final target material M@GHSP_2_, obtained through hydrophobic modification of GHSP_2_, were placed in an 80 °C oven for 24 h. Changes in mass and volume were tracked before and after heating. As shown in Figure S11, neither sample exhibited any noticeable changes in appearance, mass, or volume after thermal treatment, demonstrating the exceptional stability and safety of the cryogels.

To endow the cryogel with high hydrophobicity for its use in building materials, a simple vapor deposition process of MTMS was conducted. The enhanced hydrophobicity resulted from the formation of a siloxane network, specifically through the establishment of Si─O─Si and Si─CH_3_ bonds [42]. Water droplets stained with methylene blue provided a more intuitive comparison of changes in surface wettability before and after modification (Figure 3f). Prior to modification, GHSP_2_ exhibited strong hydrophilicity, with water droplets fully penetrating the cryogel within 2 s (Figure S12). Following MTMS modification, M@GHSP_2_ demonstrated excellent hydrophobicity. The initial water contact angle (WCA) of water droplets on the material surface was approximately 130°. As the droplet residence time increased, the WCA eventually stabilized around 120° (at 275 s) (Figure 3g and Video S1). This behavior is also related to changes in surface roughness of the network structure following vapor deposition [43]. Furthermore, the self‐cleaning performance of M@GHSP_2_ was tested, with contaminants swiftly washed away by simple water flushing (Figure 3 h and Video S2), restoring a clean surface. The three‐dimensional schematic diagrams illustrated the excellent cleaning capability of the modified cryogel and visually interpreted the influence of its microstructure on thermal performance (Figure 3i). This unique channel architecture not only impedes heat conduction but also facilitates efficient thermal energy storage through phase‐change emulsion particles anchored near the pores, significantly enhancing insulation performance [44, 45]. Comparative analysis revealed that M@GHSP_2_ also exhibited outstanding mechanical properties: it maintained approximately 0.82 MPa at 80% strain and possessed a high compressive strength of 16.52 MPa (Figure S13).

To elucidate the advantages conferred by the anisotropic architecture, the direction‐dependent mechanical and thermal properties of the aerogel were examined. The axial compressive strength increased from 14.47 to 16.52 MPa, whereas the radial compressive strength was 10.83 MPa, 34.4% lower than the axial value. This mechanical anisotropy is consistent with the anisotropic thermal transport discussed above: the axial thermal conductivity remained at 0.06 W m^−1^ K^−1^, while the radial value decreased to 0.035 W m^−1^ K^−1^, corresponding to a 41.7% reduction. These results indicate that the aligned porous architecture simultaneously regulates load transfer and heat transport, providing a continuous load‐bearing pathway along the freezing direction while enhancing thermal resistance in the radial direction. Such direction‐dependent properties enable application‐specific orientation of the aerogel, with the radial direction favoring thermal insulation and the mechanically stronger axial direction providing structural support.

All the performance characteristics described above provide a solid foundation for the application of the aerogel in building envelopes, radiative‐cooling panels, thermal‐protective layers, and cold‐chain packaging, particularly under increasingly harsh and variable outdoor conditions.

### 3.4 PRC Performance: Characterization and Outdoor Testing

Superior PRC performance was observed in the GHS cooler with nano‐silica relative to its GH counterpart, as manifested by the 1.2% increase in solar reflectance and a concurrent boost in infrared emissivity. This enhancement is primarily ascribed to the efficient backscattering of solar light and emission of thermal infrared radiation enabled by the three‐dimensional Si─O─Si network of SiO_2_ [46]. The additional improvement in the spectral properties of the cryogel cooler with the addition of PTP was attributed to a synergistic effect between SiO_2_ and the phase‐change emulsion, which effectively scattered sunlight, thereby increasing the cryogel's reflectance [32]. Attributable to the denser siloxane network formed by MTMS modification, which extended the optical path, the M@GHSP_2_ cooler exhibited optimal solar reflectance (95.28%) and infrared emissivity (97.04%). Theoretically, this effective reflection of solar radiation is expected to lower the surface temperature, thus achieving efficient cooling. All the data mentioned above are presented in Figure 4a,b and Table S2.

**FIGURE 4:**
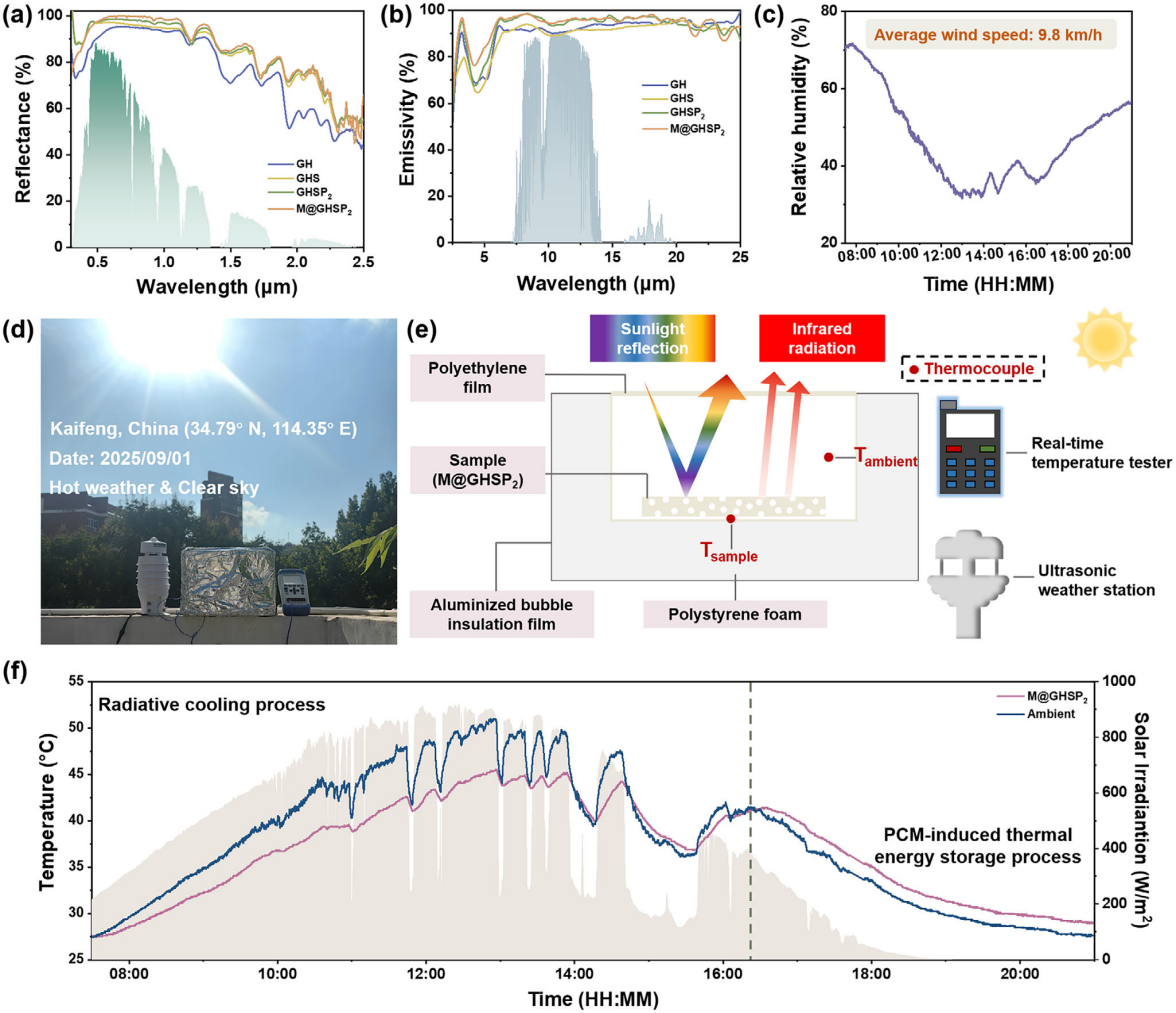
PRC performance and outdoor application demonstration. (a) Spectral UV‐visible‐infrared reflectance of four cryogel coolers presented against the AM1.5 solar spectra. (b) Spectral emittance of four cryogel coolers presented against the atmospheric transparent window. (c) Time‐dependent humidity curve on the test day. Real‐condition testing: (d) physical diagram and (e) schematic diagram of the outdoor testing device. (f) Curves of solar radiation, the real‐time temperature of the sample, and the environment during outdoor testing.

Outdoor testing was conducted under real‐world conditions to evaluate the PRC performance of the cryogel cooler, during which the ambient temperature, relative humidity, and average wind speed were monitored. Figure 4c revealed a distinct diurnal pattern in humidity: high in the morning, dropping to a daily minimum (approximately 33% RH) during peak solar irradiation (12:00–14:00), and rising again after sunset. The average wind speed throughout the day was 9.8 km h^−1^. To maximize the demonstration of the optimal sample (M@GHSP_2_) in real outdoor conditions, it was tested during summer in Kaifeng (34.79 °N, 114.35 °E), a city in China known for its high temperatures. The prevailing weather conditions and the setup of the outdoor testing apparatus are illustrated in Figure 4d. Additionally, a schematic diagram was created to illustrate the device's internal structure, as shown in Figure 4e. The apparatus consists of a sample chamber, a real‐time temperature tester, and a compact ultrasonic weather station. To minimize heat conduction and convection, the sample chamber was constructed from polypropylene foam wrapped with aluminum foil, and its surface was covered with a polyethylene film that serves as a sunlight‐transmitting layer. A K‐type thermocouple was affixed to the back of the sample using copper tape to record its temperature variations, and an additional temperature probe was deployed to monitor the ambient temperature inside the cavity.

Based on the comprehensive analysis of the systems, the M@GHSP_2_ cooler was selected as the subject for outdoor testing, compared against ambient temperature from 7:30–21:00, as shown in Figure 4f. The full‐day variation curves of the sample temperature (T) and ambient temperature (T_amb_) clearly exhibited four characteristic stages, visually revealing the dynamic thermal management process enabled by the synergistic cooperation among PRC, PCM, and the cryogel matrix:

#### 3.4.1 Stage 1. Sub‐Ambient Cooling and Diurnal Energy Storage

During this phase, T_amb_ continuously rose with the increasing intensity of solar radiation. Notably, T consistently remained lower than T_amb_, indicating the sample's effective PRC capability. This is because the cellulose cryogel matrix and the functional SiO_2_ nanoparticles loaded on its network surface possess high solar reflectivity, reflecting a large portion of the solar radiation. Simultaneously, their high emissivity in the atmospheric window (8–13 µm) enables continuous heat radiation into the cold outer space [47]. Under natural sunlight, the M@GHSP_2_ cooler exhibited a distinct temperature plateau lasting nearly 3 h (11:43–14:36), achieving remarkable PRC performance. Furthermore, the observed temperature difference (ΔT) between T and T_amb_ in the curve reached a maximum cooling temperature of approximately 6.2°C. Specifically, this plateau is attributed to the storage of solar energy during the melting phase transition of PW. The PCM absorbs and stores a significant amount of latent heat of fusion during the solid‐liquid phase change, thereby suppressing the sample's temperature rise. Under natural sunlight irradiation, PW melts as the solar radiation intensity increases. By noontime (12:23), when the daily maximum solar radiation intensity reached 912 W m^−2^, the solar energy was also maximally stored within the sample.

#### 3.4.2 Stage 2. Dynamic thermal equilibrium

From 14:44 to 16:26, the T and T_amb_ curves nearly overlap, indicating that the sample entered a dynamic thermal equilibrium. This is attributed to two factors: the decrease in solar irradiance brings the sample's PRC power and the heat absorbed from the environment into balance; concurrently, the melting process of PW is essentially complete during this stage, resulting in saturated heat storage capacity and no longer providing significant heat absorption buffering. Consequently, the sample's temperature evolution is jointly governed by the environmental heat load and the PRC power, resulting in a synchronized variation with the ambient temperature.

#### 3.4.3 Stage 3. Lagged Cooling and Thermal Energy Release

At dusk (∼16:26), the solar irradiance dropped to 365 W m^−2^. Correspondingly, the T and T_amb_ curves intersected, marking the onset of the release of stored latent heat (indicated by the gray dashed line in Figure 4f). Subsequently, as solar radiation gradually diminished and T_amb_ decreased rapidly, the cooling rate of T was noticeably slower than that of T_amb_, resulting in T remaining consistently higher than T_amb_. This phenomenon clearly revealed the thermal energy release process of PW. The latent heat stored in the PCM during the daytime began to be released as the ambient environment cooled, providing a persistent internal heat source for the sample. This process slowed the cooling rate and exhibited a significant thermal lag [48]. This characteristic is crucial for delaying the nocturnal temperature drop and mitigating diurnal temperature fluctuations.

#### 3.4.4 Stage 4. Phase Transition Plateau and Exothermic Solidification

During the nighttime period (20:00–21:00), a distinct temperature plateau emerged in the T curve, which was a characteristic signature of PCM solidification. As T fell below the solidification point of PW, a liquid‐to‐solid phase transition occurred, accompanied by the release of its latent heat of solidification. The released heat effectively counterbalanced the heat loss from the sample to the environment via radiation and convection, thereby maintaining a constant T for a certain duration. ΔT_max_ of up to 2.4°C was observed in this stage. In summary, the M@GHSP_2_ cooler demonstrated significant energy‐saving potential through its dynamic switching between cooling and thermal‐buffering modes.

To elucidate the role of the PCM, we performed heat‐transfer simulations using COMSOL Multiphysics 6.3 with the measured ambient temperature and solar irradiance from the actual test day (Table S3). We examined two critical groups: M@GHSP_2_ (with PCM) and M@GHS (without PCM). The simulated real‐time temperature curves and their difference revealed three distinct stages (Figure S14a). During the morning warming period (8:40–10:55), PCM melting absorbed latent heat, keeping M@GHSP_2_ cooler than M@GHS. Around midday (10:55–15:05), once the sample temperature exceeded the phase‐transition temperature and the PCM had fully melted, the two samples exhibited comparable thermal behavior and their temperature curves overlapped. During the evening cooling period (16:30–19:30), PCM solidification released stored latent heat, making M@GHSP_2_ warmer than M@GHSP. The temperature difference curve further illustrates these results, showing differences of −4.29°C in the morning and +3.85°C in the evening (Figure S14b). These results clearly demonstrate that the PCM acts as a thermal buffer, reducing temperature fluctuations and confirming the synergistic effect between radiative cooling and phase‐change energy storage.

### 3.5 Energy Efficiency: Simulation and Analysis

The net cooling power of the samples was further evaluated through numerical simulations, with the non‐radiative heat transfer coefficient, *h_c_*, set to 0, 4, 8, and 12 W m^−2^ K^−1^. Without considering the latent heat associated with the phase transition (Figure 5a,b), M@GHS exhibited a maximum theoretical daytime net cooling power of 69 W m^−2^ at (*ΔT* = 0), where its surface temperature was equal to the ambient temperature. Under nighttime conditions, the absence of solar heat gain increased the net cooling power to 83 W m^−2^. After incorporating the latent‐heat contribution of the phase‐change material, the net cooling power and 8 h‐equivalent cooling capacity of M@GHSP_2_ are shown in Figure 5c,d. Once the surface temperature exceeded the phase‐transition temperature (33°C), a distinct step‐like change in the net cooling power emerged. During the daytime, melting of the phase‐change material absorbed thermal energy, resulting in an overall increase of 10.21 W m^−2^ in the 8 h‐ equivalent average cooling power. Conversely, at night, solidification released the stored latent heat, leading to a corresponding decrease of 10.21 W m^−2^ in the equivalent cooling capacity.

**FIGURE 5:**
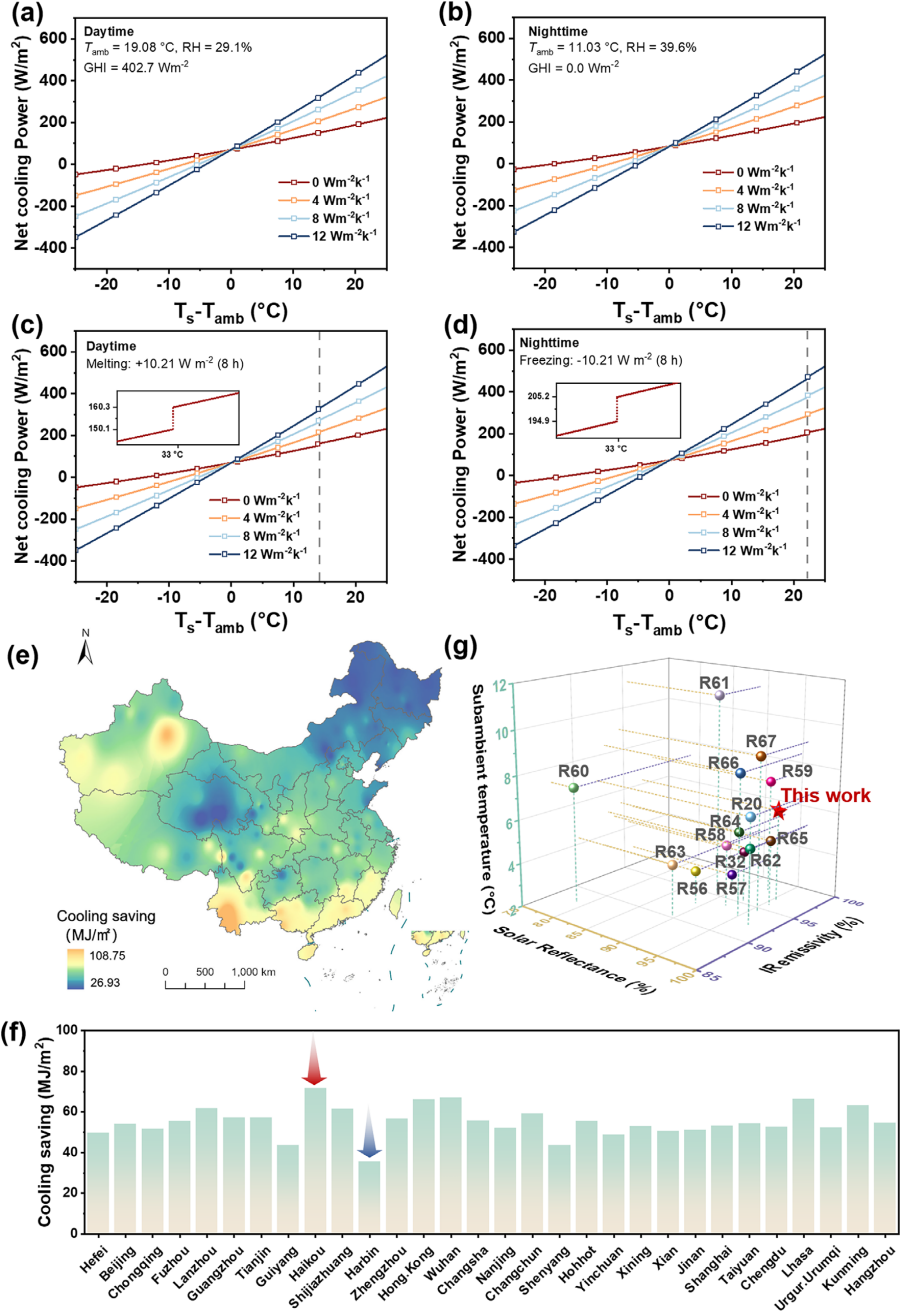
Cooling performance and energy efficiency simulation. Calculated net cooling power of the M@GHS for different non‐radiative heat transfer coefficients (*h*_*non* − *radiative*_) at the specific ambient temperature in (a) daytime and (b) nighttime. Calculated net cooling power of the M@GHSP_2_ for different *h*_*non* − *radiative*_ at the specific ambient temperature at (c) daytime and (d) night‐time (The dashed line indicates the phase transition temperature of PTP, ≈33°C). (e) Energy‐saving potential of M@GHSP_2_ across China based on cooling energy consumption. (f) Building energy‐saving performance simulation results of 30 cities in China. (g) Comparative analysis of our key performances, including solar reflectance, IR emissivity, and sub‐ambient temperature, with existing studies [20, 32, 56, 67].

In this study, EnergyPlus 23.2.0 was used to analyze a small office‐building model. Specific parameters are shown in Table S4. The model accounted for internal loads from equipment, occupants, and lighting, with cooling schedules adjusted to reflect realistic operational conditions. Energy balance equations for both interior and exterior building surfaces were solved through hourly iterations performed six times per time step. Weather data were obtained from the online repository provided by the U.S. Department of Energy. Energy consumption (EC) is influenced by geographic and climatic conditions, including solar radiation, humidity, cloud cover, and ambient temperature [49, 50]. Therefore, EC simulations were conducted for nearly 300 cities across China, covering major climate zones, with 30 representative cities selected for detailed analysis.

Figure 5e revealed significant regional variations nationwide, showing that energy‐saving potential was closely related to the distribution of solar irradiance and ambient temperature. Among them, Haikou, a representative low‑latitude city, achieved the highest daily average cooling energy of approximately 71.55 MJ·m^−2^, while Harbin, located at a high latitude, exhibited a notably lower value of only 35.54 MJ·m^−2^ (Figure 5f). Theoretically, this geographical dependency confirmed that the synergistic effect of PRC and phase‑change energy storage was most effective in cities with large diurnal temperature variations. By mitigating intense solar heat gain and buffering day‑night temperature fluctuations, the material significantly reduced building cooling loads, thereby contributing to substantial energy savings. Moreover, the performance of the cooler fabricated was benchmarked against the literature. The M@GHSP_2_ here developed demonstrates significant advantages across three key aspects: solar reflectivity, infrared emissivity, and daytime sub‐ambient cooling temperature, as shown in Figure 5g and Table S5.

Equally important toward scalability, sustainability, and practical implementation are the global efforts to decarbonize the built environment [51]. Besides operational emissions (heating, cooling, lighting), embodied carbon (materials like concrete, steel, and insulation) is of paramount relevance. In this context, standards such as the Whole Life Carbon Assessment (WLCA) aim to account for whole‐life carbon emissions, manage carbon budgets, reduce life cycle emissions, and deliver a net‐zero future for the built environment. Strategies to reduce embodied carbon include replacing high‐footprint thermal management materials derived from petroleum, such as extruded polystyrene (EPS), expanded polystyrene (XPS), or polyurethane foams (PUR). In this context, Environmental Footprint Declarations for the built environment reveal embodied carbon values of 4.03, 2.68, 4.56, or 3.36 kg of CO_2_ equivalents for 1 m^2^ of stone wool, EPS, XPS or PUR. On the contrary, cellulosic materials are recognized for their environmental benefits, with a footprint as low as 0.47 kg of CO_2_ equivalents for 1 m^2^ [52]. For a more evident comparison, we applied the life cycle assessment (LCA) methodology [53], to calculate the *cradle‐to‐gate* climate change potential of the material here developed utilizing state‐of‐the‐art databases recently updated (ecoinvent v3.12, released in November 2025), coupled with the Environmental Footprint v3.1 life cycle impact assessment methodology. Given the laboratory‐scale fabrication, the energy consumption is set aside as its incorporation could distort the results. Based on the life cycle inventory provided in Tables S6 and S7, which are provided for the sake of transparency, a climate change of 2.81 kg CO_2_ equiv. kg^−1^ is obtained, where gelatin and methyltrimethoxysilane have the largest share with 34.3% and 28.1%, respectively. Interestingly, this climate change value is even below of that shown by benchmark materials such as polystyrene or thermoplastic polyurethane foams [54]. This low footprint is translated into carbon payback periods (the time needed for operational energy savings to offset the carbon emissions created by manufacturing and installation) of months for biobased thermal‐management materials, as opposed to a few years needed for non‐renewable materials. Thus, with its cellulosic content and role as a passive thermal management material, which further accelerates carbon footprint offsets in the use phase, the cryogels here developed have a huge potential to render remarkably reduced carbon payback periods [55].

## 4 Conclusion

Here we designed a temperature‐adaptive biomimetic and biobased cryogel that integrates conventional PRC with phase‐change functionality. The porous scaffold provides an ultralow radial thermal conductivity of 0.035 W m^−1^ K^−1^), creating multiple effective optical pathways; while the aligned channels deliver high solar reflectance (95.28%) and strong infrared emissivity (97.04%) in the atmospheric window. The embedded phase‐change emulsion droplets within the GA/HEC/SiO_2_ network enable adaptive and dynamic temperature regulation. Under sunlight, the cooler reaches a maximum sub‐ambient cooling of ∼6.2°C; at night, it remains ∼2.4°C above ambient, preventing overcooling. These results confirm the potential of this composite cryogel for performance‐managed, temperature‐adaptive thermal regulation in passive radiative cooling applications. This PRC‐integrated phase‐change cryogel system shifts from passive adaptation to active thermal management using low‐footprint materials as derived from life cycle assessment results. By coupling spectral regulation with spatiotemporal heat transfer, this material offers a more sustainable platform for next‐generation low‐energy buildings and related applications.

## Author Contributions

**Sining Huang**: performing the experiments, data curation, and writing – original draft preparation. **Zhiguo Li**: software, resources. **Yang Liu**: visualization. **Chengyu Wang**: supervision, software. **Yi Lu**: writing – review & editing and supervision; **Erlantz Lizundia**: writing – review & editing, visualization, and funding acquisition. **Siqi Huan**: writing – review &editing, visualization, supervision, methodology, funding acquisition, and conceptualization.

## Conflicts of Interest

The authors declare no conflicts of interest.

## Supporting information




**Supporting File 1**: advs78323‐sup‐0001‐SuppMat.docx.

**Supporting File 2**: advs78323‐sup‐0002‐VideoS1.mp4.

**Supporting File 3**: advs78323‐sup‐0003‐VideoS2.mp4.

## Data Availability

Data will be made available on request.
