# Supplementary material for: Biobased Boston Ivy‐Inspired Cryogel Integrating Radiative Cooling and Phase Change Energy Storage for Adaptive Thermal Regulation
Source: Adv Sci (Weinh). 2026 Oct 15:e78323. Online ahead of print. doi: 10.1002/advs.78323 (PMC13672683; doi:10.1002/advs.78323)
Supplement: Supplementary file 1 [file ADVS-9999-e78323-s002.docx]

*Supporting Information for*

**Biobased Boston Ivy-Inspired Cryogel Integrating Radiative Cooling and Phase Change Energy Storage for Adaptive Thermal Regulation**

*Sining Huang^a,b,c^, Zhiguo Li^c^, Yang Liu^c^, Chengyu Wang^a,c^, Yi Lu^d*^, Erlantz Lizundia^e*^ Siqi Huan^a,c*^*

^a^ State Key Laboratory of Utilization of Woody Oil Resource, Northeast Forestry

University, Harbin, China.

^b^ Horticultural Branch, Heilongjiang Academy of Agricultural Sciences, Harbin, Heilongjiang, 150069, P.R. China

^c^ Key Laboratory of Bio-based Material Science & Technology (Ministry of Education), Northeast Forestry University, Harbin, P.R. China.

^d^ Institute of Process Engineering, Chinese Academy of Sciences, Beijing 100190, P. R. China

^e^ Life Cycle Thinking Group, Department of Graphic Design and Engineering Projects, Faculty of Engineering in Bilbao, University of the Basque Country (EHU), 48013 Bilbao, Spain

*** Corresponding authors:**

Siqi Huan, [siqi.huan@nefu.edu.cn](mailto:siqi.huan@nefu.edu.cn)

Erlantz Lizundia, [erlantz.liizundia@ehu.eus](mailto:erlantz.liizundia@ehu.eus)

Yi Lu, [luyi@ipe.ac.cn](mailto:luyi@ipe.ac.cn)

**Table of contents (Supporting Information)**

**S1. Supplementary Notes**

Supplementary notes 1: calculation of solar reflectance and infrared emissivity.

Supplementary notes 2: calculation of cooling power during the day and night.

**S2. Supplementary Characterizations**

Supplementary Characterization of PTP and Anisotropic Cryogel.

**S3. Supplementary Figures**

Supplementary Figure S1-Figure S14.

**S4. Supplementary Table**

Supplementary Table S1-Table S7.

**S5. Antifouling Performance**

Supplementary Video S1-Video S2.

**S1. Supplementary Notes**

**Supplementary notes 1:** calculation of solar reflectance and infrared emissivity.

The average solar reflectance ($\overline{R}_{solar}$) is defined as a weight average of reflectance:

…………………….……...(Supporting Equation 1)

In Supporting Equation 1, $R(\lambda)$is the reflectance of the cryogels, $I_{solar}(\lambda)$ is the AM 1.5 Global solar irradiance.

The average atmospheric window emissivity ($\overline{\varepsilon}_{LWIR}$) is obtained from a weight average of the emissivity between 8 and 13 μm with respect to the atmospheric transparent window:

…………………………….……. (Supporting Equation 2)

In Supporting Equation 2, $I_{BB}(\lambda,T)$ is the spectral intensity emitted by a blackbody at temperature$T$. $\varepsilon(\lambda)$ is the surface’s angular spectral thermal emissivity in the range of 8-13μm. $I_{BB}(\lambda,T)$is calculated by Planck’s law:

$I_{BB}(\lambda,T)=\frac{2c^{2}h}{\lambda^{5}}\frac{1}{e^{\frac{hc}{\lambda k_{B}^{T}}}-1}$……………………………………(Supporting Equation 3)

where $h$is the Planck constant, $c$is the speed of light in a vacuum, $k_{B}$is the Boltzmann constant. In this work, the temperature $T$is 298 K.

$\varepsilon(\lambda)$is the surface thermal emissivity at wavelength$\lambda$, which is calculated via following equation:

$\varepsilon(\lambda)=A(\lambda)=1-R(\lambda)-T(\lambda)$………………………………(Supporting Equation 4)

where $A(\lambda)$ is the absorptivity and $T(\lambda)$ is the transmittance.

**Supplementary notes 2:** calculation of net cooling power during the day and night.

A steady‑state energy balance model was established to quantify the net radiative cooling power of the samples. Under the assumption that the sample is exposed to ambient conditions with a surface temperature $T_{\text{s}}$and an air temperature $T_{amb}$, the net cooling power can be calculated using the following energy balance equation:

$$P_{net}(T_{s})=P_{rad}(T_{s})-P_{atm}(T_{amb})-P_{sun}-P_{non-radiative}$$

…………………………...……………………………………..(Supporting Equation 5)

Considering the effect of PCM, we extend the net cooling power as:

$$P_{net}(T_{s})=P_{rad}(T_{s})-P_{atm}(T_{amb})-P_{sun}-P_{non-radiative}+P_{PCM}$$

…………………………...……………………………………..(Supporting Equation 6)

where $P_{rad}(T_{s})$ represents the thermal radiation power provided by the sample, which can be expressed as:

$$P_{rad}(T_{s})=\int\int_{0}^{\infty} [\varepsilon_{s}(\lambda,\theta)I_{BB}(\lambda,T_{s})]d\lambda\cos\theta d\Omega$$

………………………………………………………………….(Supporting Equation 7)

where is the angle integral over a hemisphere, and $\varepsilon_{s}(\lambda,\theta)$ is the emissivity of the sample at the wavelength $(\lambda)$ and direction $(\theta)$. $I_{BB}(\lambda,T_{s})$ is the spectral radiation of the sample and its value can be calculated via equation in section 4

where $P_{atm}(T_{amb})$ is the inward radiative work of heat from the surrounding atmosphere and is calculated as:

$$P_{atm}(T_{amb})=\int\int_{0}^{\infty} [\varepsilon_{s}(\lambda\text{，}\theta)\varepsilon_{\text{atm}}(\lambda,\theta)I_{BB}(\lambda,T_{amb})]d\lambda\cos\theta d\Omega$$

………………………………………………………………….(Supporting Equation 8)

where$\varepsilon_{amb}(\lambda,\theta)=1-t(\lambda)^{1/\cos\theta}$ is the emissivity of the atmosphere at the wavelength $(\lambda)$ and direction $(\theta)$, and $t(\lambda)$ is the transmittance of the atmosphere at the wavelength $(\lambda)$ and in the zenith direction. These data can be selected from MODTRAN 4.

$P_{sun}$ is energy power from the sun and is calculated as:

$P_{sun}=\int_{0}^{\infty} [\varepsilon_{s}(\lambda,\theta_{sun})I_{AM1.5}(\lambda)]d\lambda$………………………..…….(Supporting Equation 9)

Non-radiative heat power $P_{non-radiative}$, including heat conduction and convection, calculated as:

$P_{con+\mathrm{cov}}=h_{non-radiative}(T_{amb}-T_{s})$……………..……………...….(Supporting Equation 10)

where $h_{non-radiative}$ is the non-radiative heat transfer coefficient. We chose values 0, 4, 8 and 12 m^-2^ K^-1^ in the calculation.

The cooling power of the PCM during daytime is ascertained when the ambient temperature exceeds 33 ℃. The calculation of $P_{PCM}$ is calculated via:

$P_{PCM}=\frac{\Delta H\times M}{T\times A}$……………………………………………(Supporting Equation 11)

where $\Delta H$ is the phase change enthalpy of PCM. We stipulated that the PTP has a phase change enthalpy of 235.32 J/g. M/A denotes the PTP dosage per unit area. Based on the sample dimensions used in the outdoor test (Figure 4), M/A is 0.125 g/cm². T is the working time of PCM. We set the average working time as 8 hours, whether in the daytime or nighttime. We stipulated that the PTP finishes the phase change from solid to liquid in the daytime and from liquid to solid at night to maximize the effect of PCM. **^[1]^**

Turpan was selected as a representative application scenario. For daytime conditions, the ambient temperature, relative humidity, and solar irradiance were fixed at 19.08 °C, 29.1%, and 402.7 W m^-2^, respectively. For nighttime conditions, the ambient temperature and relative humidity were set to 11.03 °C and 39.6%, respectively, with no solar irradiation. The above spectra of humidity and temperature were also obtained from MODTRAN 5.

**S2. Supplementary Characterizations**

**Characterization of PTP and anisotropic cryogel**

*Droplet Sizing.* A static light scattering device (Mastersizer 3000, Malvern Panalytical, UK) was used to analyze the mean droplet diameter and size distribution of Pickering emulsions containing varying oil phase volumes. The refractive indices used in the analysis were 1.45 for beeswax and 1.33 for water. The Sauter mean diameter (*D_32_*) was employed to quantify the average droplet size of each sample, calculated using the formula *D_32_*=*Σn_i_d_i_^3^/Σn_i_d_i_^2^*, where *n_i_* denotes the number of droplets with diameter *d_i_*. After 24 hours of storage in lab environment, all measurements were conducted in triplicate to ensure accuracy.

*Emulsion Morphology.* The morphology of emulsion droplets during storage was observed using an optical microscope (MP41, Mshot, China) with a 10X objective lens. A 5 µL sample was placed on a slide and covered with a glass coverslip.

*Fourier transform infrared spectroscopy (FTIR).* FTIR (Thermo-Fisher, USA) characterized chemical functionalities across 400-4000 cm^-1^ spectral range with 32 accumulative scans. Pre-analysis desiccation of samples and detector components minimized atmospheric interference.

*Rheological Behavior*. The rheological behavior of the emulsion containing different oil types was measured using a rheometer (AR2000ex, TA Instruments) with parallel plates and a fixed gap of 1 mm. Measurements were conducted across a shear rate range from 0.01 to 100 s^-1^, and angular frequencies from 0.1 to 100 rad s^-1^, all at 25 ℃.

*Mechanical Properties*. Compressibility of phase change foam was evaluated by computerized electronic universal testing machine (Suns, UTM2503, China). A compression mold with a range of 5 kN was used for the test, and the compression was stopped when the deformation of the object reached 90%.

*Thermal Properties.* The thermal behavior of phase change foams was characterized using differential scanning calorimetry (DSC) (DSC 8000, PerkinElmer, USA). BTC foam samples were heated from 20 °C to 80 °C at a rate of 10 °C min^-1^.

*Brunauer-Emmett-Teller (BET).* The specific surface area and pore size distribution were analyzed using the BET (JW-BK132F, JWGB, China) method via nitrogen adsorption isotherms obtained at -196℃.

*PRC performance*. The outdoor thermal behavior of the samples was simulated using the Heat Transfer Module in COMSOL Multiphysics 6.3. Each sample was simplified as a 4 × 4 × 1.8 cm rectangular cuboid. The mesh contained approximately 2.7 × 10⁵ tetrahedral elements with an average element size of about 1 mm. The time step was 60 s, and the relative tolerance was 10^-6^. Thermodynamic, optical, and phase-change parameters were taken from experimental measurements. The simulation used the measured outdoor ambient temperature and solar irradiance from the test day. Because the sample was wrapped in aluminum foil and insulating foam during outdoor testing, the bottom and side surfaces were treated as adiabatic. To clarify the effect of phase change, two simulations were performed under identical boundary conditions, one including latent heat and one excluding it. Comparison of the two results clarifies the temperature-regulating role of the phase-change material.

**S3. Supplementary Figures**

**Figure S1.** Demonstration of bionic approaches for cryogel cooler in microstructure and dynamic temperature regulation mechanisms.

**Figure S2.** Schematic diagram of the PTP preparation process.

**Figure S3.** (a) Schematic diagram of the structure and encapsulation mechanism of phase-change emulsions via Pickering emulsion template. (b) Scanning electron microscope image and schematic diagram of PTP particles. (c) Optical microscope image of PTP. (d) Size distribution of PTP and its visual appearances (inset images). (e) G’ versus G’’ plotted as a function of oscillation frequency of PTP.

**Figure S4.** Schematic diagram of preparing anisotropic cryogels via directional freezing.

**Figure S5.** Surface scanning electron microscope image of (a) GHSP_0.5_, (b) GHSP_1_ and their corresponding magnified pictures. Cross- section scanning electron microscope image of (a_1_) GHSP_0.5_ and (b_1_) GHSP_1_.

**Figure S6.** FTIR of GH, GHS, GHSP_2_ and M@GHSP_2_.

**Figure S7.** Thermal conductivity. (a) Directional Freezing and (b) Non-Directional Freezing.

**Figure S8.** (a) Porosity of GH, GHS, GHSP_x_ and M@GHSP_2_. (b) Pore size distribution of M@GHSP_2_.

**Figure S9.** Thermal stability analysis via (c) TGA and (d) DTG for GHSP_0.5_ and GHSP_1_.

**Figure S10.** (a) DSC thermograms showing heating-cooling cycles for PTP and anisotropic cryogels with different PTP contents. (b) Latent heat of anisotropic cryogels with different PTP contents. (c) Thermal cycling stability of M@GHSP_2_ demonstrated through 1 and 100th cycle DSC profiles.

**Figure S11.** Leak-proof test. (a) Visual integrity assessment of GHSP_2_ and M@GHSP2 before/after 24 h at 80℃. Time-dependent (b) mass and (c) volume changes at elevated temperature.

**Figure S12.** Captured images of water contact angle testing for GHSP_2_.

**Figure S13.** Mechanical properties of four anisotropic cryogels: (a) Stress-strain curves and (b) compressive strength.

**Figure S14.** Simulation of outdoor thermal behavior for samples with and without PCM using COMSOL Multiphysics 6.3 Heat Transfer Module. (a) Real-time diurnal temperature curves of the M@GHS and M@GHSP_2_. (b) Real-time Δ*T* curve between M@GHS and M@GHSP_2_.

**S4. Supplementary Table**

**Table S1.** Parameters for thermal cycling stability of M@GHSP_2_

| Cycle number | *T_m_* (℃) | *ΔH_m_* (J/g) | *T_c_* (℃) | *ΔH_c_* (J/g) |
| --- | --- | --- | --- | --- |
| 1 | 29.83 | 67.02 | 23.43 | 43.53 |
| 100 | 29.47 | 63.25 | 23.59 | 40.33 |

**Table S2.** Spectral properties of four cryogel coolers.

| Sample | $\overline{R}_{solar}(\%)$ | $\overline{\varepsilon}_{LWIR}(\%)$ |
| --- | --- | --- |
| GH | 92.0 | 91.29 |
| GHS | 93.20 | 91.34 |
| GHSP_2_ | 94.46 | 96.20 |
| M@GHSP_2_ | 95.28 | 97.04 |

**Table S3.** Information of the building model applied in energy analysis.

| **Items** | **Specifications** |
| --- | --- |
| Density | 62.5 kg/m^3^ |
| Thermal conductivity | 0.035 W m^-1^ K^-1^ |
| Specific heat capacity | 1800 J kg^-1^·K^-1^ |
| Solar reflectance | 95.28% |
| IR emissivity | 97.04% |
| Average phase change temperature | 29.5℃ |
| Phase change temperature range | 7 K |
| Latent heat | 67.02 J g^-1^ |

**Table S4.** Information of the building model applied in energy analysis.

| **Items** | **Specifications** |
| --- | --- |
| Building prototype | Small office building |
| Interior design temperatures | Summer: 26 ℃ |
| Lighting density | 10.65 W m^-2^ |
| Equipment power density | 7.64 W m^-2^ |
| Occupants | 0.0565105297 person m^-2^ |
| Roof/wall/door construction | M@GHSP_2_-Metal Fe-Roof Insulation-Metal Fe/M@GHSP_2_-MetalFe-8IN Concrete HW-Wall Insulation-Wood Board/ Metal Fe-Roof Insulation-Metal Fe |
| Metal Fe | Thermal conductivity: 45W m^-1^ K^-1^  Density: 7842 kg m^-3^  Specific heat: 500 J kg^-1^ K^-1^ |
| 8IN Concrete | Thermal conductivity: 1.73 W m^-1^ K^-1^  Density: 2243 kg m^-3^  Specific heat: 837 J kg^-1^ K^-1^ |
| Roof Insulation | Thermal conductivity: 0.049 W m^-1^ K^-1^  Density: 256 kg m^-3^  Specific heat: 836 J kg^-1^ K^-1^ |
| Wall Insulation | Thermal conductivity: 0.0432 W m^-1^ K^-1^  Density: 91 kg m^-3^  Specific heat: 837 J kg^-1^ K^-1^ |
| Wood Board | Thermal conductivity: 0.1 W m^-1^ K^-1^  Density: 608 kg m^-3^  Specific heat: 1630 J kg^-1^ K^-1^ |
| M@GHSP_2_ | IR emissivity:0.9704  Solar reflectance:0.9528 |

**Table S5.** Comparison parameters with other researches.

| Reference | Solar reflectance (%) | IR emissivity (%) | Sub-ambient temperature (℃) |
| --- | --- | --- | --- |
| R20 | 97.2 | 92.3 | 6.8 |
| R32 | 92.6 | 95.5 | 4.3 |
| R56 | 89.0 | 93.0 | 3.5 |
| R57 | 89.3 | 90.4 | 4.3 |
| R58 | 92.0 | 94.0 | 4.8 |
| R59 | 95.9 | 93.4 | 5.1 |
| R60 | 78.5 | 88.0 | 7.3 |
| R61 | 92.8 | 98.2 | 7.2 |
| R62 | 90.3 | 96.0 | 2.9 |
| R63 | 88.8 | 95.5 | 11.2 |
| R64 | 92.8 | 94. 7 | 5.4 |
| R65 | 95.6 | 94.6 | 9.0 |
| R66 | 94.8 | 93.1 | 8.4 |
| R67 | 95.6 | 95.9 | 5.0 |
| This work | 95.28 | 97.04 | 6.2 |

**Table S6.** Material inventory for LCA.

| **Item** | **Amount (g)** | **Source/provider** |
| --- | --- | --- |
| Hydroxyethyl cellulose ^a^ | 81.10 | carboxymethyl cellulose production, powder \| carboxymethyl cellulose, powder \| Cutoff, U - RoW |
| Gelatin ^b^ | 162.20 | glycine production \| glycine \| Cutoff, U - RoW |
| Parafin wax | 259.30 | paraffin production \| paraffin \| Cutoff, U - RoW |
| TOCNF | 3.10 | Own process, see Table S2 |
| PVA ^c^ | 4.70 | vinyl acetate production, from ethylene acetoxylation \| vinyl acetate \| Cutoff, U - RoW |
| SiO2 ^d^ | 324.10 | activated silica production \| activated silica \| Cutoff, U - GLO |
| Methyltrimethoxysilane (166.70 grams) ^e^ | 255.05 | tetraethyl orthosilicate production \| tetraethyl orthosilicate \| Cutoff, U - GLO |
| Water | 16800.00 | water production, deionised \| water, deionised \| Cutoff, U - RoW |
| Evaporated water | 16800.00 | Emission to air |
| Process wastes | 1.20 | process-specific burdens, sanitary landfill \| process-specific burdens, sanitary landfill \| Cutoff, U - CH |
| **1000.00 grams** | | **Process output** |

^a^: a proxy material was used as hydroxyethyl cellulose is absent in the used database. Carboxymethyl cellulose is also a non-toxic water-soluble cellulose derivative.

^b^: a proxy material was used. Gelatin is composed of amino acids, including glycine, proline, and hydroxyproline.

^c^: a proxy material was used. Vinyl acetate is used to polymerize polyvinyl acetate and then hydrolyze this into PVA.

^d^: a proxy material was used to account for the additional processing of silica sand into the hydrophilic fumed silica with a specific surface area of 200 m²/g used in this work.

^e^: a proxy material was used. TEOS is used as both a liquid silicon alkoxides, and produced through alcoholysis of a chlorosilane precursor. 1.53 kg of TEOS per kilogram of MTMS is used to represent the molar quantity of silicon.

**Table S7.** Material and energy inventory for 1 kg of “TEMPO-CNF” processing by TEMPO-Oxidation and Homogenization according to Ref **^[2]^**. Energy consumption is estimated according to Refs **^[3]^** and **^[4]^**.

| **Comment** | **Item** | **Amount (kg)** | **Source/provider** |
| --- | --- | --- | --- |
| Source material | Cellulose | 1.060 | sulfate pulp production, from softwood, bleached \| sulfate pulp, bleached \| Cutoff, U - RoW |
| Catalyst, 0.1 mmol·g^-1^ | TEMPO ^a^ | 0.023 | piperidine production \| piperidine \| Cutoff, U - RoW |
| Co-catalyst, 1 mmol·g^-1^ | Potassium bromide ^b^ | 0.164 | market for potassium chloride, industrial grade \| potassium chloride, industrial grade \| Cutoff, U - GLO |
| Oxidant, 3.5-5 mmol·g^-1^ | 3.5 kg NaClO at 10 wt% | 0.350 | market for sodium hypochlorite, without water, in 15% solution state \| sodium hypochlorite, without water, in 15% solution state \| Cutoff, U - RoW |
|  |  | 3.150 | water production, deionised \| water, deionised \| Cutoff, U - RoW |
| - | 44 mL, 4 M NaOH | 0.00704 | market for sodium hydroxide, without water, in 50% solution state \| sodium hydroxide, without water, in 50% solution state \| Cutoff, U - RoW |
|  |  | 0.0436 | water production, deionised \| water, deionised \| Cutoff, U - RoW |
| - | 35 mL, 12 M HCl | 0.01531 | market for hydrochloric acid, without water, in 30% solution state \| hydrochloric acid, without water, in 30% solution state \| Cutoff, U - RoW |
|  |  | 0.0261 | water production, deionised \| water, deionised \| Cutoff, U - RoW |
| For the reaction and washing | Water | 113.000 L | water production, deionised \| water, deionised \| Cutoff, U - RoW |
| Pulping, stirring, refining, homogenization | Electricity | 20.000 kWh | market group for electricity, medium voltage \| electricity, medium voltage \| Cutoff, U - CA |
| Wastewater | | 16.1936 | treatment of wastewater, average, wastewater treatment \| wastewater, average \| Cutoff, U - CA-QC |
| TEMPO-CNF  (1.0450 mmol_COOH_/g_TOCNF_) | | 1.000 | Process output, dry CNFs |

^a^ used as a proxy material. Piperidine, it is the exact saturated N-heterocycle found in TEMPO.

^b^ used as a hybrid proxy material. It accurately reflects the mining and processing impacts of potassium.

**References**

[1]	M. Yang, H. Zhong, T. Li, B. Wu, Z. Wang, and D. Sun, "Phase Change Material Enhanced Radiative Cooler for Temperature-Adaptive Thermal Regulation", *ACS Nano*, *17* (2023), 1693, <https://doi.org/10.1021/acsnano.2c11916>.

[2]	P. Gallo Stampino, L. Riva, C. Punta, G. Elegir, D. Bussini, and G. Dotelli, "Comparative Life Cycle Assessment of Cellulose Nanofibres Production Routes from Virgin and Recycled Raw Materials", *Molecules,* 26 (2021), 2558, <https://doi.org/10.3390/molecules26092558>.

[3]	F. Piccinno, R. Hischier, S. Seeger, and C. Som, " From laboratory to industrial scale: a scale-up framework for chemical processes in life cycle assessment studies", *J. Cleaner Prod.***,** *135 (2016)*, 1085, https://doi.org/10.1016/j.jclepro.2016.06.164.

[4]	R. Arvidsson, D. Nguyen, and M. Svanström, "Life Cycle Assessment of Cellulose Nanofibrils Production by Mechanical Treatment and Two Different Pretreatment Processes", *Environ. Sci. Technol.*, *49* (2025), 6881, <https://doi.org/10.1021/acs.est.5b00888>.
